# Structural Quantification of the Surface-Confined Metal-Organic Precursors Simulated with the Lattice Monte Carlo Method

**DOI:** 10.3390/molecules28104253

**Published:** 2023-05-22

**Authors:** Jakub Lisiecki, Paweł Szabelski

**Affiliations:** Department of Theoretical Chemistry, Institute of Chemical Sciences, Faculty of Chemistry, Maria Curie-Skłodowska University, Pl. M.C. Skłodowskiej 3, 20-031 Lublin, Poland; jakub.lisiecki@mail.umcs.pl

**Keywords:** 2D porous networks, adsorption, Ullmann coupling, functional molecules, Monte Carlo simulations, polymerization

## Abstract

The diversity of surface-confined metal-organic precursor structures, which recently have been observed experimentally, poses a question of how the individual properties of a molecular building block determine those of the resulting superstructure. To answer this question, we use the Monte Carlo simulation technique to model the self-assembly of metal-organic precursors that precede the covalent polymerization of halogenated PAH isomers. For this purpose, a few representative examples of low-dimensional constructs were studied, and their basic structural features were quantified using such descriptors as the orientational order parameter, radial distribution function, and one- and two-dimensional structure factors. The obtained results demonstrated that the morphology of the precursor (and thus the subsequent polymer) could be effectively tuned by a suitable choice of molecular parameters, including size, shape, and intramolecular distribution of halogen substituents. Moreover, our theoretical investigations showed the effect of the main structural features of the precursors on the related indirect characteristics of these constructs. The results reported herein can be helpful in the custom designing and characterization of low-dimensional polymers with adjustable properties.

## 1. Introduction

The spontaneous creation of low-dimensional molecular structures on surfaces has been recently studied to develop and optimize strategies for the fabrication of nanomaterials with predefined properties [1,2,3,4,5]. Among these, diverse quasi-1D polymers comprising covalently bonded organic monomers have been recognized as very promising with respect to their potential applications in nanoelectronics and related areas [6,7,8,9]. In this case, two predominant types of polymeric structures have been fabricated and characterized experimentally, including molecular wires [9,10] and graphene nanoribbons [8,11]. A useful method to obtain these structures has been the surface-assisted Ullmann coupling reaction in which halogenated, usually (poly)aromatic hydrocarbon (PAH), molecules are adsorbed on catalytically active surfaces of such metals as copper, silver, and gold in ultra-high vacuum (UHV) conditions. Upon adsorption, and often with additional heating, the halogen atoms are scised, and the resulting highly reactive aryl radicals recombine to form persistent covalent polymers [12,13,14,15]. The above strategy has been successfully used to create polyphenyl wires and carbon nanoribbons having multiple architectures as well as other constructs, such as networks [13,16,17] and ring oligomers [18,19].

One of the main challenges in the optimization of the on-surface synthesis of covalent polymers has been the selection of suitable monomeric units able to spontaneously form an adsorbed superstructure with presumed properties (e.g., connectivity, periodicity, ramification, porosity, etc.). To that end, molecular parameters such as size, shape, and intramolecular distribution of halogen substituents can be tuned to direct the self-assembly and polymerization toward a desired product. Moreover, it has also been shown that metallic substrates having special anisotropic structural features (e.g., grooves) can be effectively used to confine the on-surface polymerization and promote the unidirectional growth of organic chains [20,21,22]. Another problematic question related to the quality of the polymers on surfaces is defect propagation. As has been observed in numerous experimental studies, the strong covalent bonding of monomers eliminates error correction in a growing polymer and self-healing of this construct [23,24]. As the monomers cannot break the erroneous connections and adopt more energetically favourable configurations, the defective outcome is petrified, so further structural modifications are hardly possible. However, in some instances, there has been observed the beneficial precursor effect that can be effectively utilized to reduce/remove completely the aforementioned drawback. Specifically, in such cases, the actual covalent linkage of monomers is preceded by the formation of a metal-organic precursor in which the coordination bonds are labile enough to allow for error correction. When this intermediate structure is further heated, the metal-organic bonds are converted into covalent ones, and a final, usually more ordered, product is obtained. An important, advantageous property of the precursor is that its architecture is usually transmitted to the corresponding covalent polymer. This effect has been observed, for example, in the formation of chains by molecules of 4,4″-dibromo-p-terphenyl on Cu(111) [25], ring oligomers of 4,4″-dibromo-mterphenyl on the same substrate [26] or 2D networks of polyphenyl molecules equipped with alkynyl bromide groups, adsorbed on Au(111) [27]. For that reason, understanding the factors that govern structure formation at the metal-organic stage of the Ullman coupling as well as controlling them play a key role in the on-surface synthesis of low-dimensional organic polymers.

As the optimal choice of the isomer to be polymerized is often not straightforward in practice, complementary theoretical methods can be helpful and more efficient. This refers especially to the synthesis of the tectons and the qualification of their on-surface performance in UHV. In the latter case, the self-assembly process and its tecton-dependent outcomes can be modelled using computer simulations with Molecular Dynamics (MD) [28,29,30,31] and Monte Carlo (MC) [32,33,34,35,36] techniques. The above methods allow for quick changes in intrinsic molecular features (size, shape, functionality, and interaction pattern) and observation of the corresponding effects induced in the adsorbed phase. This efficiency has been particularly visible in the coarse-grained models, where the simplified discrete representation of both interacting species and adsorbing surface were used. For example, the coarse-grained MC calculations were revealed to be accurate and effective in predicting various molecular superstructures (especially networks) sustained by hydrogen bonding [37], metal-organic ligand coordination [34,38,39], halogen bonding [40], or even van der Waals interactions [41].

Recently we demonstrated that MC modelling can also be successfully applied to the precursor self-assembly in the surface-assisted Ullman coupling of aryl halides. With this methodology, we were able to classify the extensive series of PAH isomers with respect to the type of precursor architecture they formed in the simulations (networks, strings, ladders, oligomers, etc.) [42,43,44,45,46]. In this contribution, we focus on the quantitative characteristics of newly selected examples of metal-organic precursors created by those molecules, with particular emphasis on the quasi-1D structures, including chains and ladders. To that purpose, quantitative descriptors, such as the 1D and 2D structure factors, radial distribution functions (RDFs), and cluster size statistics, were calculated and compared, aiming at the identification of the main similarities and differences between the modelled systems. Our main objective here was to investigate how to modify monomer properties in order to create low-dimensional precursors (and thus subsequent covalent connections) with predefined properties. Moreover, the results of this contribution explain the relationship between the architecture of these precursors and the quantitative measures that can be applied (also experimentally) to characterize them.

## 2. Results and Discussion

**Figure 1:**
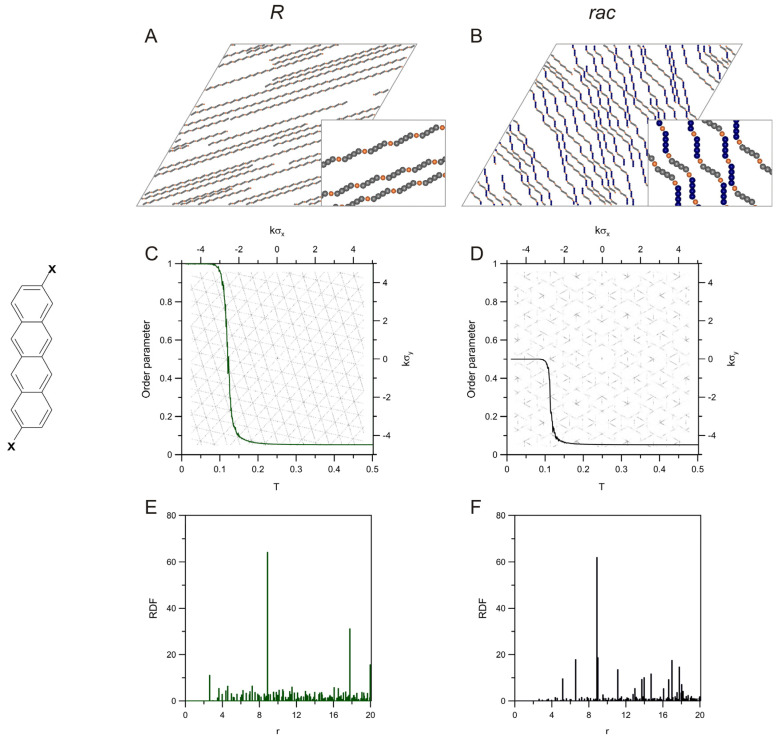
Snapshots of the enantiopure (**A**) and racemic (**B**) overlayers comprising molecules of **t28** (sketched on the left of panel **C**), 400 *R* (grey linkers) and 200 *R* + 200 *S* (grey + blue linkers), respectively, mixed with 400 metal atoms (orange adatoms). Panels (**C**,**D**) present the effect of temperature on the orientational order parameter calculated for these systems. The background images in (**C**,**D**) show the corresponding 2D-dimensional structure factors. The plots in panels (**E**,**F**) are the metal-metal radial distribution functions calculated for the enantiopure and racemic self-assembly of **t28**; Final temperature *T* = 0.01, surface coverage *θ* = 0.05.

Let us start this section with the simulated precursors, which were characterized by the lowest structural complexity. In the following, we will present adsorbed systems with a gradually decreasing degree of order and discuss the related quantitative descriptors. In the examples selected here, we tried to account for the various representative types of structure formation encountered previously for the analogous PAH units [42,43,44,45,46]. Our main objective was to examine how these different self-assembly scenarios affect the associated functional dependencies. To that purpose, a set of new monomeric units was prepared, enabling estimation of the effect of size/shape and halogenation pattern on the calculated descriptors. Figure 1 shows a snapshot of the structurally simplest metal-organic assembly that was obtained for the enantiomer *R* of 2,8-dihalotetracene (**t28**).

For comparative purposes, analogous results predicted for the corresponding racemic mixture, *rac*-**t28**, were also displayed in the figure. As can be seen in panel (*A*), the molecules of **t28**(*R*) formed long straight chains aligned in parallel and running along one common direction. These unidirectional strands were fully periodic; that is, the contributing monomers were always uniformly oriented relative to the propagation direction. This type of uniform alignment enabled the growth of an energetically favourable set of the longest possible chains in which the number of terminal, undercoordinated fragments was minimized. The average length of these chains was equal to 32.21 molecules, as determined from the chain length distribution function shown in Figure S1. Let us emphasize that the above quantity (absolute value) is, in general, dependent on the system size and, thus, it has only comparative meaning in the current context. Accordingly, as for each of the tectons, the simulations were performed for the same *L* = 200, so it was possible to determine the relative effect of molecular properties on the average size of the corresponding aggregates.

When the enantiomer *S* of **t28** was introduced to the adsorbed overlayer, a noticeable change in the geometry of the resulting metal-organic constructs took place. In this case, the molecules created mixed chains with a bent shape and random sequences of the contributing units *R* and *S.* Similarly to the enantiopure self-assembly, the molecules attempted to create long chains, which, however, were no longer parallel. This effect induced noticeable winding of the mixed strings, which resulted in a larger average chain length (61.81) in comparison with **t28**(*R*) (see Figure S1). As can be noticed in panel (B), parallel fragments of these chains occasionally occurred for the longer enantiopure sequences comprising a few neighbouring molecules of *R* or *S* (grey and blue fragments, respectively). Moreover, contrary to **t28**(*R*), the metallic centres sustaining the mixed chains did not form straight lines, and these chains were aperiodic in terms of composition and geometry. The results obtained for the isomer **t28** were structurally similar to their counterparts simulated previously for the smaller analogue 2,6-dihaloantracene (**a26**) [42,43,44,45,46]. Recently, the architecture of the enantiopure and racemic chains of **a26,** which we predicted theoretically, was also confirmed experimentally using the homocoupling of 2,6-dibromoanthracene on a Au(111) surface [47]. Those experimental results demonstrated that the particular directionality of metal-ligand bonding assumed for the model linear PAHs correctly represents the pattern of interactions occurring in the real precursor structures.

To quantify the differences between the enantiopure and racemic self-assembly of **t28**, in Figure 1, we showed the effect of temperature on the order parameter *δ* calculated for these distinct cases and plotted the associated metal-metal radial distribution functions (RDFs, see the Supplementary Materials). The order parameter *δ* provides information on the extent of unidirectional alignment of linear molecular backbones along three main directions but has a somewhat different interpretation for the nonlinear PAH tectons (see the Supplementary Materials for definition). Nevertheless, this parameter is equal to one in systems in which all molecules (or linear fragments comprising the lowest locant in bent molecules) are oriented in the same direction; values of *δ* close to zero mean equal probability of each of the possible orientations. The results shown in panel (C) indicate the complete unidirectional ordering of **t28**(*R*), which occurred when the temperature decreased below ~0.12. Above this temperature, which corresponds to the onset of structural transition and chain formation, the adsorbed overlayer was a collection of randomly oriented molecules (i.e., in the three main directions of the lattice) and unbounded metal atoms, so that *δ* was very small—close to 0.1. For the racemic mixture, similarly, a sharp increase of the order parameter was observed at ~0.12, however, reaching only 0.5 (see panel D). This value is a direct consequence of the two dominant molecular orientations in the overlayer of *rac-***t28**. Specifically, all of the enantiomers *R* therein are oriented in one direction, while the enantiomers *S* are rotated by 60 degrees relative to this direction. Because the enantiomers are equally abundant, according to Equation (S1) in the Supplementary Materials, the parameter δ equals 0.5 for this particular system.

Panels (E) and (F) of Figure 1 present the metal-metal RDFs calculated for the enantiopure and racemic overlayers of **t28**, respectively. As can be seen in the first of these panels, the RDF exhibits characteristic maxima (highest peaks) at about 9 and 18. These features reflect the periodic arrangement of metal atoms in the enantiopure chain, which are separated by $\sqrt{79}\approx 8.89$. Accordingly, the distance between a selected metal atom and its successive neighbours in the chain are integer multiples of $\sqrt{79}$ (two first values are represented in the plot). For the racemic RDF, the highest peak is centred at $\sqrt{79}$ and larger multiples of this value contribute much less intensively. This effect means a shorter range of ordering, which corresponds to the distance between two metal atoms attached to one molecule of **t28**. Note that this intermetallic distance is the same for *R* and *S* so that in any sequence of the enantiomers (*R*-*S*, *R*-*R*, and *S*-*S*), the shortest spacing between metal atoms is equal to $\sqrt{79}$. For larger distances, the chance of meeting a metal atom within the chain is strongly dependent on the actual enantiomeric sequence of the chain. In consequence, many low peaks appear in the racemic RDF at the larger values of *r*.

The background images in panels (C) and (D) present the 2D structure factors (theoretical diffraction patterns) calculated for the corresponding **t28**-based precursors. The triangular grid of straight lines observed for the enantiopure chains results from the three possible orientations these chains can adopt on the surface (differing by 60 degrees, imposed by the symmetry of the adsorbing lattice). As the obtained images are averages over ten system replicas, all of the orientations are represented, each by a series of parallel lines. The straight chains of **t28**(*R*) can be treated as one-dimensional diffraction gratings with spacing equal to the shortest intermetallic distance ($\sqrt{79})$, generating the linear diffraction pattern seen in panel (C). When the disturbances in the chain periodicity take place, like for the racemate from panel (D), the diffraction lines are no longer continuous, but some of their fragments vanish so that the resulting pattern is more complex.

**Figure 2:**
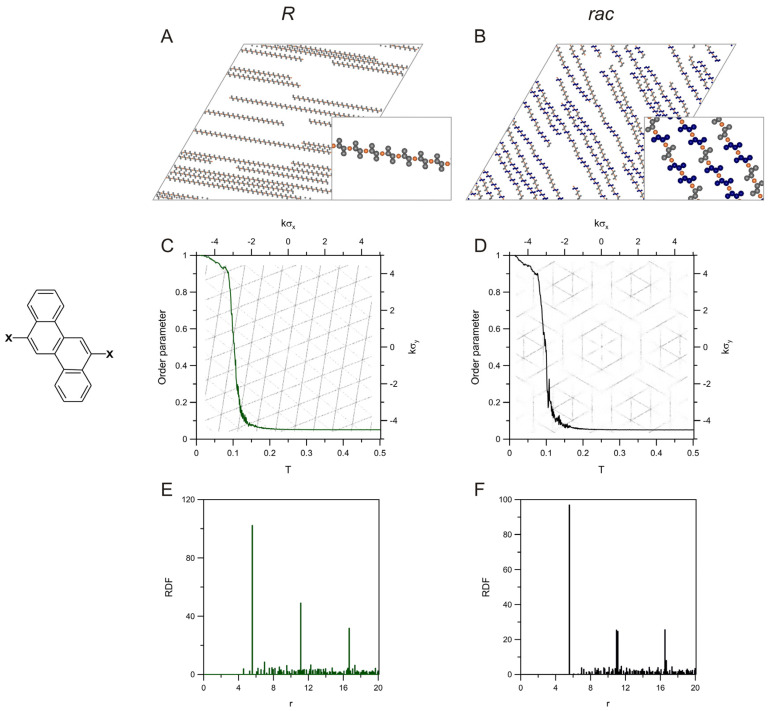
Snapshots of the enantiopure (**A**) and racemic (**B**) overlayers comprising molecules of **c28** (sketched on the left of panel **C**), 400 *R* and 200 *R* + 200 *S*, respectively, mixed with 400 metal atoms. Panels (**C**,**D**) present the effect of temperature on the orientational order parameter calculated for these systems. The background images in (**C**,**D**) show the corresponding 2D-dimensional structure factors. The plots in panels (**E**,**F**) are the metal-metal radial distribution functions calculated for the enantiopure and racemic self-assembly of **c28**; *T* = 0.01, *θ* = 0.05.

The next examples, related to the haloderivatives of 2,8-chrysene (**c28**) and 3,9-phenenthrene (**p39**), show how the shape and size of the PAH building blocks with the same directionality of interactions affect the precursor self-assembly and the corresponding structural descriptors. Figure 2 presents the results obtained for the first tecton having bent geometry but containing the same number of benzene rings as the halogenated tetracene isomer **t28** from Figure 1. The chains created by **c28** (*R*) were straight and oriented along one direction, which provided maximum space for their extended growth. In this case, the average chain length was equal to 43.78 (see Figure S2), which is larger than for **t28**(*R*), which resulted directly from the shorter fragment of **c28** involved directly in the chain linkage (2 and 4 segments for **c28** and **t28**, respectively). As can be seen in panel (A), the molecular segments not involved in the metal-organic bonding protrude from the chains making these structures effectively wider as compared to **t28**(*R*). The unidirectional alignment of the chains can also be seen in panel (C); that is, the orientational order parameter was close to one when the temperature approached zero. The periodic architecture of these chains was also reflected in the corresponding RDF shown in panel (E). The highest peaks occurred therein for *r* equal to $\sqrt{31}$ (closest intermetallic separation in the chain) and for larger integer multiples of this value. The diffraction pattern predicted for the enantiomer *R* of **c28** was shown in panel (F), where the triangular grid of lines can be seen—similarly to **t28** (see Figure 1C). However, in the case of c28(*R*), a less dense mesh was obtained, which corresponds to the smaller distance between metal atoms within the chains.

The racemic self-assembly of **c28** produced the mixed chains shown in panel (B). These long chains, even though bent, run along one common direction. Note that because of the shorter fragment, which separates active centres in a molecule of **c28** (two segments), and the presence of the protruding side segments, the resulting chains were less winded, as compared to the tetracene unit from Figure 1. The structures shown in panel B of the figure were in full agreement with the experimental results obtained recently for the Ullmann coupling of 6,12-dibromochrysene on the Au(111) surface [48]. The average chain length calculated for *rac*-**c28** was equal to 29.46 (see Figure S2). This effect, contrary to **t28**, demonstrated the shortening of the chains when the other enantiomer (**c28**(*S*)) appeared in the adsorbed overlayer. In the case of the order parameter presented in panel (D), the simulations predicted a sharp increase of this structural descriptor at the temperature ~0.15, similar to the enantiopure overlayer of **c28**. Note that at low temperatures, the curve from panel (D) reaches about one, which corresponds to the uniform orientation of the contributing enantiomers *R* and *S*. As can be easily noticed in panel (B), the middle two-segment long parts of the molecules *R* and *S* are rotated by 120 degrees relative to each other, which induces bending of the chains. However, despite this effect, all of the molecular arms remain parallel, and, according to the adopted definition of *δ*, the resulting value should be equal to one—in agreement with the dependency from panel (D).

The RDF obtained for the racemic mixture *rac*-**c28** (see panel F) was qualitatively very similar to its enantiopure counterpart. Here, the effect already observed for **t28** can also be noticed; that is, the position of the main peaks of the racemic RDF is associated with the sequence of the *R*/*S* units in a given chain. Because the shortest intermetallic distance is not dependent on this sequence (i.e., it is equal to the distance between the active centres of either *R* or *S*), the highest peak occurs at $r=\sqrt{31}$, and farther impulses at the larger multiples correspond to the homochiral periodic sequences of *R* and *S*. However, like for *rac*-**t28**, these impulses (especially at 2$\sqrt{31}$) are accompanied by the nearby peaks reflecting the mixed enantiomeric composition and the (slight) bending of the chains. Panel (D) shows the corresponding diffraction pattern, which is structurally simpler as compared to the one calculated for **t28**. In this case, the less bent chains of *rac*-**c28** generated the more ordered (linear) arrangements of the metallic centres, resulting in the characteristic hexagonal zones in the background image in panel (D).

**Figure 3:**
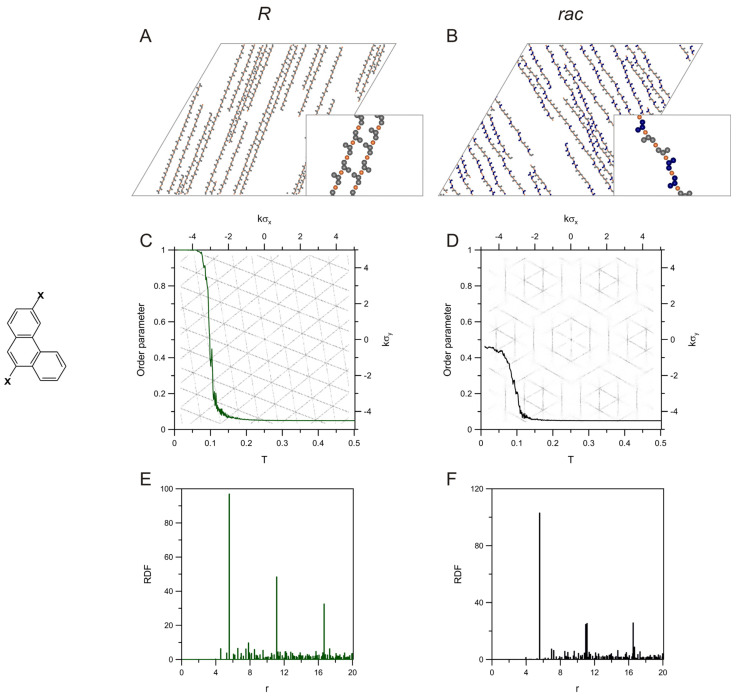
Snapshots of the enantiopure (**A**) and racemic (**B**) overlayers comprising molecules of **p39** (sketched on the left of panel **C**), 400 *R*, and 200 *R* + 200 *S*, respectively, mixed with 400 metal atoms. Panels (**C**,**D**) present the effect of temperature on the orientational order parameter calculated for these systems. The background images in (**C**,**D**) show the corresponding 2D-dimensional structure factors. The plots in panels (**E**,**F**) are the metal-metal radial distribution functions calculated for the enantiopure and racemic self-assembly of **p39**; *T* = 0.01, *θ* = 0.04.

For comparative purposes, in Figure 3, we presented the results obtained for the second smaller analogue, **p39**, consisting of three benzene rings fused into a bent shape. As can be noticed in the figure, the simulated outcomes are, in general, very similar to those obtained for the bigger molecule **c28**. This refers to the morphology of the adsorbed overlayers (panels (A) and (B)) as well as to the quantitative descriptors shown in parts (C)–(F). Note that the position and height of the main peaks of the RDFs calculated for the enantiopure and racemic systems comprising **p39** are nearly identical to their counterparts calculated for **c28**. This effect comes directly from the presence of the same fragment in **p39** and **c28**, which consist of two segments and separates a pair of metal atoms in the corresponding chains by $\sqrt{31}$. It is also evident that the lack of symmetric protruding segments in the chains formed by the smaller isomer **p39** did not seriously affect the way in which these chains are packed on the surface. On the other hand, slight shortening of the chains in *rac*-**p39** occurred (although not as evident as for **c28**); that is, the average chain length decreased from 38.60 (*R*) to 35.60 (*rac*) (see Figure S3). The obtained contact value of the order parameter, ~0.5, results from the two possible orientations of the straight parts of *R* and *S* in the chain. However, in this case, unlike for **c28**, the straight fragments relevant to *δ* comprise the segments that are both involved in the bonding (chain-forming parts of *R* and *S* are rotated by 120 degrees). For that reason, according to the adopted definition of *δ* (see the Supplementary Materials), for *rac*-**p39,** the value of this parameter at low temperatures should be equal to 0.5, in agreement with the results from panel (D). To construct polymeric chain structures comprising phenanthrene units, molecules such as 2,7-dibromophenanthrene on a Cu(111) surface have been used in the experiment, and the resulting metal-organic precursors bonding motifs analogous to **p39** were found [49].

**Figure 4:**
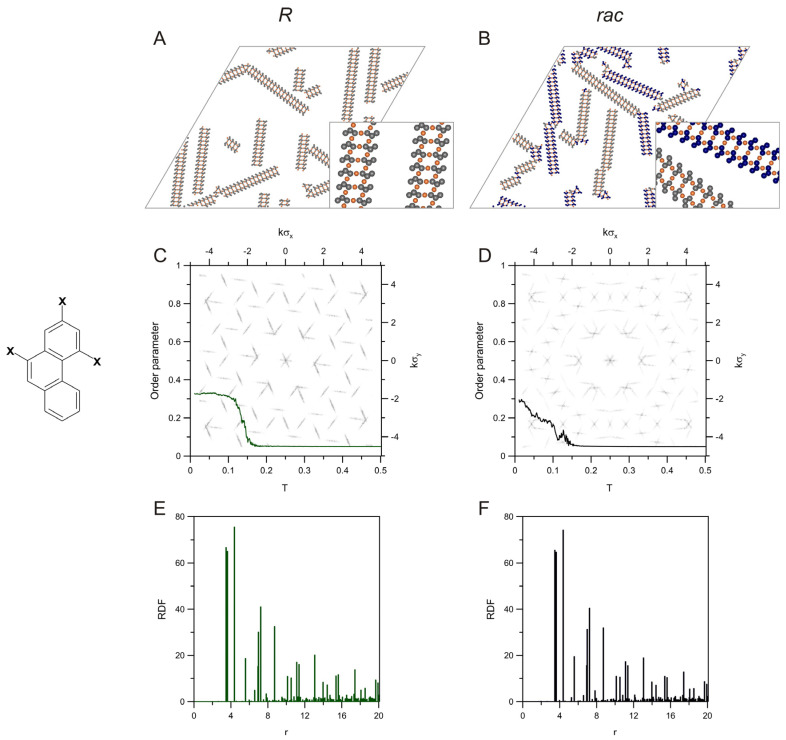
Snapshots of the enantiopure (**A**) and racemic (**B**) overlayers comprising molecules of **p24_10** (sketched on the left of panel **C**), 400 *R*, and 200 *R* + 200 *S*, respectively, mixed with 600 metal atoms. Panels (**C**,**D**) present the effect of temperature on the orientational order parameter calculated for these systems. The background images in (**C**,**D**) show the corresponding 2D-dimensional structure factors. The plots in panels (**E**,**F**) are the metal-metal radial distribution functions calculated for the enantiopure and racemic self-assembly of **p24_10**; *T* = 0.01, *θ* = 0.045.

Our simulations showed that certain PAH isomers, apart from the ability to form the chain structures from Figure 1, Figure 2 and Figure 3, can also create ladders structures, such as those shown in panel (A) of Figure 4. In this case, the molecules of 2,4-phenenthrene derivative, **p24_10**(*R*), formed ordered constructs in which two straight and periodic chains of oppositely oriented molecules were inter-bridged, resulting in the extended ladders. This building block was studied in our previous work, providing an interesting example of chiral resolution in adsorbed overlayers comprising ladder PAH precursors [42,43,44,45,46]. For that reason, we included it in the set of probed tectons and calculated the associated structural descriptors (not determined before). The formation of structurally similar two-stranded ladders has also been observed experimentally in the coordination-directed on-surface polymerization of brominated porphyrins on Au(111) [50].

The average size of the ladders was equal to 30.95 (see Figure S4), which corresponds to around 15.5 bimolecular units being the measure of the length of these constructs. For the corresponding racemate, the self-assembly produced a similar precursor, in which chiral resolution occurred, as shown in panel (B). Even though in this mixed overlayer, long periodic fragments of the homochiral ladders *R* and *S* can be found, they adopted multiple discrete orientations, reducing the extent of the order. The average aggregate size calculated for this mixed system was equal to 49.06 (see Figure S4). Panels (C) and (D) of Figure 4 show the corresponding plots of the order parameter calculated for these two systems. As can be seen in the first panel, the effective formation of ladders occurs at a temperature equal to about 0.11, and it manifests in the quite intense increase of *δ*, which reaches ~0.33 when *T* goes to zero. This contact value of *δ* is consistent with the structural properties of the unidirectional ladders, which were exemplified in panel (A). As mentioned previously, according to the definition of the parameter *δ*, in each of these ladders, there is only one most abundant molecular orientation out of the three possible associated with the three growth directions of the ladders. In the simulations, we observed that the coexisting differently oriented ladders occurred in most of the replicas. However, the formation of these constructs was often unbalanced; that is, the longest ladders usually propagated along one direction, and the two remaining ones were then less preferred, as seen in panel (A). In consequence, the order parameter given by the equation was not equal to zero (as it would be for three equally probable orientations) but considerably larger. A similar, although less steep, increase of the order parameter at low temperatures can be observed for the racemic mixture of **p24_10**, shown in panel (D). In this case, the growth of homochiral ladders in the racemate was more difficult because of the self-sorting mechanism, which required a molecule *R* (*S*) to find the corresponding seed (ladder fragment) where it could be incorporated. In consequence, at the transition temperature (around 0.15), the molecules *R* and *S* self-assembled at a slower rate when mixed. Nevertheless, the contact value of the order parameter (at T→0) obtained for the racemate was similar to that for the enantiopure overlayer, and this effect resulted from the three orientations (imposed by the three possible growth directions), which the molecules *R* and *S* could take when in the complementary homochiral ladder. Because the racemic overlayer comprised mirror-image ladder forms, the calculated RDF was nearly identical to its enantiopure counterpart. This effect can be seen when comparing the plots from panels (E) and (F). In both cases, the dominant contributions (first three peaks) occurred at $r=2\sqrt{3},\;\sqrt{13},\;$ and $\sqrt{19}$, respectively, and they correspond to the closest intermetallic distances within the basic structural motifs of the ladders.

For the enantiopure overlayer of **p24_10**(*R*), our calculations predicted the 2D structure factor that was shown in the inset to panel (C). In this case, the diffraction pattern consisted of a series of incomplete parallel lines (with sectional signal extinctions) running along the three directions (rotated by 60 degrees relative to each other). The anisotropic character of the ladders, manifested by the parallel diffraction lines, in combination with the finite width of these structures, was the source of the additional features present in the plots. In the case of the racemic mixture of **p24_10**, the plot shown in panel (D) comprised more reflexes as compared to part (C), and this effect appeared due to an additional set of orientations available for the homochiral ladders. Specifically, the closest angular distance between a pair of ladders, *R* and *S,* was equal to around 21 degrees. In consequence, the ladders could grow along 6 possible directions (3 for *R* and 3 for *S*) and produce the more complex diffraction pattern (hexagonal concentric rounded contours can be seen in panel (F), indicating increased disordering).

**Figure 5:**
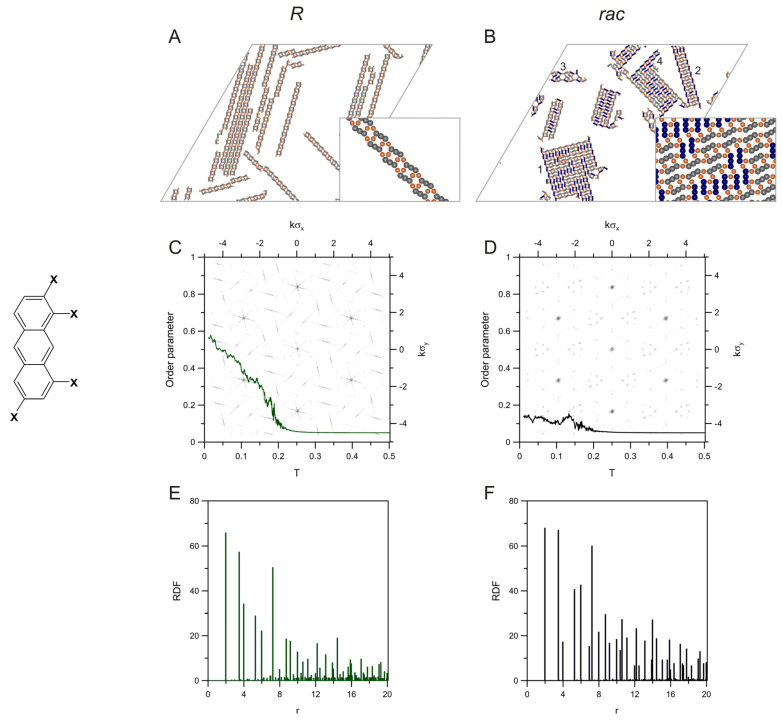
Snapshots of the enantiopure (**A**) and racemic (**B**) overlayers comprising molecules of **a1268** (sketched on the left of panel **C**), 400 *R*, and 200 *R* + 200 *S*, respectively, mixed with 800 metal atoms. Panels (**C**,**D**) present the effect of temperature on the orientational order parameter calculated for these systems. The background images in (**C**,**D**) show the corresponding 2D-dimensional structure factors. The plots in panels (**E**,**F**) are the metal-metal radial distribution functions calculated for the enantiopure and racemic self-assembly of **a1268**; *T* = 0.01, *θ* = 0.05.

The chiral resolution of enantiomers described above was not the only scenario observed for the ladder-forming molecules of our study. Figure 5 shows the results of calculations performed for the isomer **a1268**. This tecton, when in the enantiopure overlayer (*R*), created the ladders shown in panel (A). The alignment of molecular cores occurring in these ladders is similar to that observed in the chiral graphene nanoribbons synthesized via the Ullmann coupling of 2,2′-dibromo-9,9′-bianthracene on the metallic (111) surfaces, including copper, silver, and gold [51]. The obtained elongated structures ran along the three directions, rotated by 60 degrees relative to each other, and were also partially fragmented. The average length of these ladders, 30.95, was, however, bigger as compared to **p24_10** (26.16).

A drastic change in the morphology of the adsorbed phase occurred when the other enantiomer (*S*) was introduced. Panel (B) shows the complex phase coexistence observed in the racemic overlayer of the 1,2,6,8-tetrahaloantracene **a1268**. In this case, at least four different local structures can be indicated, and these are the rectangular domains with random *R*/*S* composition, ribbons of two main types (with hexagonal pores and compact), and the small homochiral fragment of the *S* ladder (blue). A similar partial ordering with the creation of directional domains has been observed in the Ullman coupling of the H-shaped tetrabromo quinazine-based monomer on a Au(111) substrate [52]. Figure S5 presents the resulting cluster size statistics based on which the average size of an aggregate formed in *rac*-**a1268** was determined to be equal to 49.06. This increased value demonstrates the increased tendency of the enantiomers of **a1268** to form extended compact domains.

The aforementioned structural differences between the enantiopure and racemic self-assembly can also be seen in the corresponding plots of the order parameter shown in panels (C) and (D). The contact value obtained for **a1268**(*R*) reached about 0.6, and this resulted from the strongly preferred alignment of the ladders in one direction as compared to the two remaining ones. For the racemate, this value was considerably smaller, reflecting the multitude of coexisting phases in which the enantiomers *R* and *S* adopted all of the allowed orientations with similar probabilities. As can be noticed in panels (E) and (F), the main contribution to the RDFs of the discussed systems occurred for r=2, $2\sqrt{3}$ and for the integer multiplies of these distances. These two values correspond to the spacing between metal atoms linked at locants 1 and 2 and, more importantly, 1 and 9 and 7 and 9, respectively. As these distances occur in each molecule, regardless of orientation and chirality, the dominant reflexes in the associated plots of the structure factor (panels (C) and (D)) represent the resulting side of the most abundant parallelogram $2\sqrt{13}$ the unit cell (α = 88°) of the metallic pattern, marked in the inset. For the enantiopure overlayer, the diffraction pattern comprises additional features that reveal the 3-fold rotational symmetry of the ladders. This effect was obviously not observed in the disordered (or only partially ordered) racemic overlayer in which the local ordering was imposed only by the intrinsic geometric features (distribution of the active centres) of a single molecule of **a1268**.

**Figure 6:**
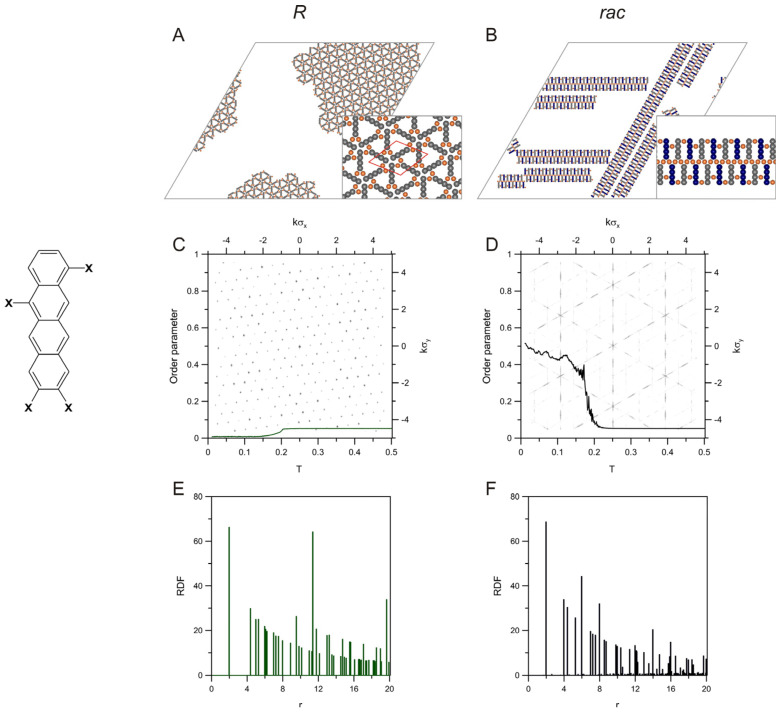
Snapshots of the enantiopure (**A**) and racemic (**B**) overlayers comprising molecules of **t1589** (sketched on the left of panel **C**), 400 *R*, and 200 *R* + 200 *S*, respectively, mixed with 800 metal atoms. Panels (**C**,**D**) present the effect of temperature on the orientational order parameter calculated for these systems. The background images in (**C**,**D**) show the corresponding 2D-dimensional structure factors. The plots in panels (**E**,**F**) are the metal-metal radial distribution functions calculated for the enantiopure and racemic self-assembly of **t1589**; *T* = 0.01, *θ* = 0.06.

A markedly different structure formation was observed in the case of the last tecton 1,5,8,9-tetrahalotetracene (**t1589**). Panel (A) of Figure 6 presents the ordered domain created by the enantiomer *R* of this (pro)chiral building block.

The obtained biporous metal-organic network comprises triangular void spaces of two sizes, and it is described by a rhombic unit cell having a side equal to $\sqrt{129}$. One characteristic motif that can be noticed in this network is the chiral *C*_3_—symmetric node in which six molecules meet and bond with six nearby metal atoms so that metal concentration is significantly increased in this local area. The formation of highly ordered covalent networks, such as the one from Figure 6A, has been infrequently met in the experiment, mainly because of various defects related to the erroneous, irreversible bonding of monomers. However, it has been shown that the larger heterotriangulene building blocks equipped with three halogen substituents are able to create extended porous networks having symmetry similar to **t1589**(*R*) [53].

The appearance of the enantiomer **t1589**(*S*) in the adsorbed overlayer resulted in a complete change of the morphology of the assembly, unlike for the string-forming tectons **t28**, **c28**, and **p39**. In this case, the double-stranded ladder structures were formed with periodic alternate sequences of the units *R* and *S*, as shown in panel (B). The ladders seen in this panel provide uniform alignment of the aromatic cores, which has often been required in the surface-assisted Ullmann synthesis of graphene nanoribbons, for example comprising 10,10′-dibromo-9,9′-bianthryl [54] and other monomeric units [55]. The average size of the simulated aggregates was equal to 56.67 molecules, as determined from the corresponding distribution function (see Figure S6). The zipper-like connection of molecular strands comprised the central metallic row providing the dense packing of metal atoms. Note that, in these racemic ladders of **t1589**, the enantiomers *R* and *S* had the same backbone orientation. In the calculations, we observed that the mixed ribbons were rarely fragmented, and they usually grew in two directions. In consequence, like for **p39**, the order parameter *δ* took values close to 0.5 at low temperatures, as demonstrated in panel (D). On the other hand, a somewhat less obvious situation takes place in the case of the enantiopure network of this isomer. As can be seen in panel (C), the order parameter vanishes when the temperature is gradually decreased, which may suggest strong disordering. In this case, however, the onset of condensation at ~0.2 corresponds to the growth of the *C*_3_—symmetric network in which only three molecular orientations are allowed, and they are equally probable, resulting in *δ* equal to zero.

The structural features of the enantiopure and racemic assemblies of **t1589** are clearly represented in the RDFs plotted in panels (E) and (F), respectively. The high peak at *r* = 2 that is seen in the first plot is directly related to the characteristic and dominant distance between a pair of metal atoms within the *C*_3_-symmetric nodal motifs sustaining the network from panel (A). The next high peak at *r* ≈ 11.35 corresponds to the side of the unit cell of this network (i.e., $\sqrt{129}$) and thus to the metal atoms (periodic images), which are placed in the neighbouring unit cells. Not that the RDF of the enantiopure network does not decay sharply, even for distances close to 20—for example, at *r* = 19.65, which confirms the highly periodic architecture of this precursor. For the ladder structures depicted in panel (F), the main contribution to the RDF, occurring at *r* = 2, comes from the central metallic rows in which the linking atoms are separated by 2 (closest distance). As it can also be noticed therein, farther peaks are centred at integer multiples of 2 (e.g., the high peaks at 6 and 8), and they create a pattern that evinces the long-range unidirectional order of the ladders.

The 2D structure factor shown in the inset to panel (C) confirms the ordered architecture of the enantiopure *C*_3_—a symmetric network of **t1589**(*R*). The predicted set of discrete spots resembles a typical diffraction pattern of ordered crystalline solids with a high degree of symmetry. On the other hand, for the highly anisotropic ladder structures comprising both enantiomers of **t1589**, we can observe fuzzed reflexes forming straight lines and corresponding mainly to diffraction on the central row of metal atoms (1D diffraction gratings, like for **t28**). The three distinct orientations of the diffraction lines correspond to the rotational degeneracy of the ladders that was imposed by the symmetry of the lattice.

## 3. The Model and Calculations

**Figure 7:**
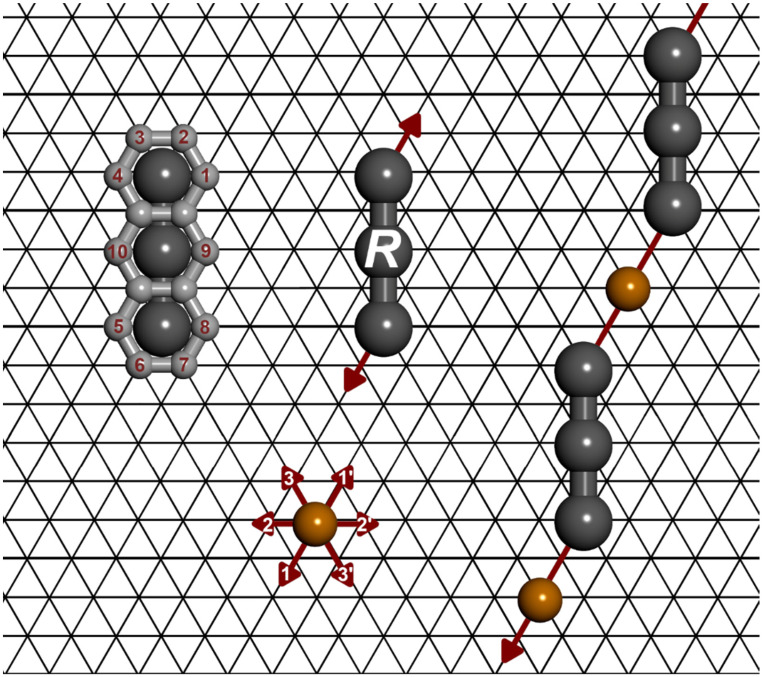
A visual representation of the simulation model using 2,6-disubstituted anthracene (**a26**(*R*)). Red arrows determine the directions of the metal-mediated intermolecular interactions corresponding to the selected substitution locations and possible colinear linker-metal-linker interactions (marked with numbers 1 and 1′ and so on).

The MC model of metal-organic self-assembly used here was the same as in our previous works [42,43,44,45,46]. Specifically, the PAH molecules studied in the following were represented by rigid connections of discrete segments, each of which corresponded to one benzene ring. These connections had different sizes and shapes (straight, bent, etc.), reflecting the actual geometry of a given molecule. In this contribution, molecules consisting of 2 (naphthalene) to 4 (tetracene, chrysene) segments were investigated. These units were adsorbed on a triangular lattice of equivalent sites with side *L*-standing for the catalytically active (111) substrate. Each molecular segment of an adsorbed molecule was allowed to occupy exactly one adsorption site (lattice vertex). To account for the halogen substituents in the considered PAH isomers, the model molecules were equipped with discrete centres of directional interactions. The number and intramolecular distribution of these centres were consistent with the halogenation pattern of a given tecton, in which the conventional IUPAC numbering of locants for substituted PAHs was used. Metal atoms mediating in intermolecular bonding were represented by single segments. Figure 7 shows an exemplary configuration of the molecules of **a26** and metal atoms co-adsorbed on the triangular lattice.

The range of the interaction between adsorbed monomers and metal atoms was limited to the nearest neighbouring sites, measured from the hexagon-based contour of a given tecton. The molecules were allowed to form single links with metal atoms, each with energy ε, or to create linear intermolecular connections comprising the mediating metal atom. The necessary condition for the latter situation to occur was the collinear alignment of the interaction directions assigned to these molecules. If this condition was satisfied, the energy of interaction was equal to 2ε. In the case of a nonlinear 120° nodal geometry, this energy was assumed to be ε due to the steric repulsion between benzene rings (segments) directly involved in the bonding. Examples of the two types of configurations contributing effectively to the net energy of the adsorbed phase are illustrated in Figure 7.

The simulations were performed on a rhombic fragment of the triangular lattice with *L* = 200. To eliminate finite-size effects, periodic boundary conditions were imposed in both planar directions. The calculations were carried out in the canonical ensemble, that is, for fixed: system size *L*, number of adsorbed components, *N*, and temperature *T*, using the conventional Metropolis sampling method [56]. The composition of the adsorbed phase was controlled by the parameters $N_{l}$ and $N_{m}$, meaning the number of molecules and metal atoms, respectively, so that $N_{l}+N_{m}=N$. The corresponding density of the overlayer, equal to the average number of segments per lattice site, was defined as: $\left(xN_{l}+N_{m}\right)/L^{2}$ where *x* is the number of segments in a given tecton. The simulations started with a set of *N* species randomly distributed over the surface. Next, a series of equilibration moves were performed to obtain the final configuration. To that end, a molecule or metal atom was picked up at random, and its potential energy in the current (old) state, $U_{o}$ was calculated based on the rules defined previously and illustrated in Figure 7. The selected component was translated to a new random position on the lattice, and in the case of a linker, the molecule was additionally rotated by a multiple of 60 degrees. If, in the new position, there was enough space (unoccupied lattice sites), the selected component was inserted therein; otherwise, it was returned to its initial position. To accept or reject the new configuration, the associated potential energy, $U_{n}$ was determined using the same scheme as for $U_{o}$ and these values contributed to the acceptance probability $p=\min\left[1,\exp\left(-\frac{\Delta U}{kT}\right)\right]$, where $\Delta U=U_{n}-U_{o}$ and *k* is the Boltzmann constant. The calculated *p* was next compared with a uniformly distributed random number $r\in \left(0,1\right).$ If r  was less than *p*, then the new configuration was accepted. In the opposite case, the selected component was left in its original place, and the whole procedure started over. The algorithmic sequence described above constitutes one MC step that was repeated many times (typically $\;N\times 10^{5}$) to equilibrate the adsorbed overlayer. Moreover, to minimize the risk of trapping the modelled systems in metastable states, the metal-organic assemblies were slowly cooled down during the simulation, that is, from T=0.51 to T=0.01 within 500 intervals of equal length. The energies and temperatures in our model were expressed in units of ε and $\left|\varepsilon \right|/k$. The quantitative results presented in the following are averages over ten independent system replicas.

All of the model halogenated PAH units discussed in this study have (pro)chiral properties; that is, these isomers can adopt mirror-image conformations when adsorbed. For that reason, two main self-assembly scenarios are possible for them, related to the enantiopure and racemic composition of the overlayer. To identify the most important similarities and differences between the structures formed under these two regimes, for each isomer, we performed separate simulations. The obtained enantiopure and racemic results were set up together to help the reader notice how the morphology and the associated structural descriptors change when both surface enantiomers of a given tecton are at play.

## 4. Summary and Conclusions

In this work, we demonstrated how the structural properties of low-dimensional metal-organic precursors on solid substrates can be controlled using halogenated building blocks with appropriate features, such as shape, size, number, and distribution of halogen atoms. The simulations performed for a few exemplary halogenated PAH units, differing by these factors, revealed that, in certain cases, slight changes in the internal structure of the monomer can produce pre-polymeric assemblies with markedly distinct properties. This refers especially to the (pro)chiral building blocks, which, apart from the enantiopure assemblies, can additionally create racemic superstructures. As we showed, in these cases, the geometry of the enantiopure and racemic overlayers can be very different, changing from network to (wide) ladder (**t1589**) and from (narrow) ladder to disordered (**p24_10**), respectively. Analogously drastic changes were, however, not observed for the single chain-forming tectons, for which the introduction of the other enantiomer usually resulted in the lack of periodicity and bending of the chains (**t28**, **c28**). In the case of the ladders, it was found that, for some tectons (**p24_10**), chiral resolution of the enantiomers can occur, producing mirror-image structures in the racemic overlayer. Interestingly, such self-sorting was not visible for the tecton **a1268**, which despite forming ladders in the enantiopure system, created a few mixed phases when in the racemate.

The metal-organic precursors reported in this work were additionally characterized by a series of structural descriptors that helped quantify the differences between these structures. To that end, we used the orientational order parameter, radial distribution functions, and one- and two-dimensional structure factors. In the case of the order parameter, it was observed that the racemic chains with two possible orientations of the contributing enantiomers were characterized by a substantially lower value (around 0.5), as compared to the enantiopure chains (close to 1)—even though in both cases the topology was the same. As we also noticed, the orientational order parameter worked fine for those systems where such highly anisotropic constructs as chains and ladders were formed, but it was less informative for the ordered networks (**t1589**). In the latter case, the value close to zero was not associated with randomness in the enantiopure overlayer but with the equally populated molecular orientations in the regular network.

The additional important structural information extracted from the simulated assemblies was the 2D-structure factor corresponding to the diffraction pattern of these systems. These indirect characteristics demonstrated how the changes in the properties of the monomer and the enantiomeric composition of the adsorbed phase affect the corresponding diffraction patterns. As we observed, the simple hexagonal lattice pattern calculated for straight chains (representing one-dimensional diffraction gratings) became partially wiped out with increasing disorder. In consequence, in the S_2D_ plots, characteristic reflexes occurred, corresponding to the shorter range of ordering in the probed assemblies. On the other hand, the periodic network of **t1589** produced the regular diffraction pattern comprising discrete spots—like for crystalline solids.

The precursor structures simulated in this work are qualitatively similar to the structures obtained previously for the other PAH tectons [42,43,44,45,46], and they can be divided based on the morphology-dependent classification used therein (strings, ladders, networks, etc.). The assemblies shown in Figure 1, Figure 2, Figure 3, Figure 4, Figure 5 and Figure 6 also illustrate the typical effects (e.g., chiral mixing vs. segregation) observed before. In this context, the latest structural descriptors introduced in this study can be indicative of entire specific classes of precursors having common morphology. Because the new PAH units created metal-organic architectures with geometric features similar to those of their counterparts reported in our previous studies, the tectons t28, c28, p39, a1268, and t1589 can be an alternative with respect to synthetic protocols and post-purification, which may be, for example, simpler and less costly.

The results of our theoretical studies can be useful in designing and optimizing molecular systems on surfaces in which labile metal-mediated structures precede the formation of persistent covalent polymers. The findings presented herein can facilitate the preliminary selection of a monomeric unit with the aim of constructing the target low-dimensional polymer having the desired structural properties. Moreover, the obtained structural descriptors, especially the 2D-structure factors, can be helpful in the quantification and comparison of the main geometric features of these metal-organic precursors. The proposed approach can be easily extended to different molecular systems comprising functional units (not necessarily PAH derivatives) and also to those interacting directionally via other relatively weak forces, such as, for example, hydrogen bonding.

## Data Availability

Data used in this paper is available to view by contacting authors.

## Supplementary Materials

The following supporting information can be downloaded at: https://www.mdpi.com/article/10.3390/molecules28104253/s1, Figure S1: Structure factors (A,B), frequency of clusters of a given size and average size of the cluster (C,D) and fractions of homo- and heterochirally bonded metal atoms (E) for enantiopure (A,C) and racemic (B,D,E) systems of t28 depicted in Figure 1; Figure S2. Structure factors (A, B), frequency of clusters of a given size and average size of the cluster (C,D) and fractions of homo- and heterochirally bonded metal atoms (E) for enantiopure (A,C) and racemic (B,D,E) systems of c28 depicted in Figure 2; Figure S3. Structure factors (A,B), frequency of clusters of a given size and average size of the cluster (C,D) and fractions of homo- and heterochirally bonded metal atoms (E) for enantiopure (A,C) and racemic (B,D,E) systems of p39 depicted in Figure 3; Figure S4. Structure factors (A,B), frequency of clusters of a given size and average size of the cluster (C,D) and fractions of homo- and heterochirally bonded metal atoms (E) for enantiopure (A,C) and racemic (B,D,E) systems of p24_10 depicted in Figure 4; Figure S5. Structure factors (A,B), frequency of clusters of a given size and average size of the cluster (C,D) and fractions of homo- and heterochirally bonded metal atoms (E) for enantiopure (A,C) and racemic (B,D,E) systems of a1268 depicted in Figure 5; Figure S6. Structure factors (A,B), frequency of clusters of a given size and average size of the cluster (C,D) and fractions of homo- and heterochirally bonded metal atoms (E) for enantiopure (A,C) and racemic (B,D,E) systems of t1589 depicted in Figure 6.
