# Supplementary material for: Structural Quantification of the Surface-Confined Metal-Organic Precursors Simulated with the Lattice Monte Carlo Method
Source: Molecules. 2023 May 22;28(10):4253. doi: 10.3390/molecules28104253 (PMC10224111; doi:10.3390/molecules28104253)
Supplement: Supplementary file 1 [file molecules-28-04253-s001.zip › molecules-2387315-supplementary.pdf]

Supplementary Materials

for

Structural Quantification of the Surface-Confined Metal-Organic Precursors Simulated with the Lattice Monte Carlo Method

Jakub Lisiecki and Paweł Szabelski*

Department of Theoretical Chemistry, Institute of Chemical Sciences, Faculty of Chemistry,

Maria Curie-Skłodowska University in Lublin,

Pl. M.C. Skłodowskiej 3, 20-031 Lublin, Poland

Order parameter

The parameter of order was calculated using the following equation:

$$\delta = \frac{|N_{l1} - N_{l2}| + |N_{l2} - N_{l3}| + |N_{l1} - N_{l3}|}{2 * N_l} \qquad \text{(Equation S1)}$$

where N_l is the total number of linkers in the system and N_{l1}, N_{l2}, N_{l3} are the average numbers of linkers oriented along the 3 main directions defined by the sections connecting two first segments of a molecule: that is the segment with the lowest locant and its nearest segment. Note that, for linear backbones (acenes) the parameter δ refers simply to the orientation of the long axis of a given molecule, while for bent molecules it describes orientation of the 2-membered fragment of their backbones. As in Equation (S1) every linker in the pair is counted twice, the result is divided by 2.

Radial function distribution

The metal-metal radial distribution function was calculated using the following formula:

$$g(r) = \frac{\langle n_r \rangle A}{N_m \pi (2rdr + dr^2)} \qquad \text{(Equation S2)}$$

where $\langle n_r \rangle$ is the average number of metal atoms in (r, $r + dr$) shell away from any given metal, $\pi(2rdr + dr^2)$ is the area of the considered shell ($dr = 0.05$), and A denotes the Cartesian area of the system ($A = L^2 * \frac{\sqrt{3}}{2}$).

1D and 2D structure factors

The one-dimensional structure factor was calculated using the following formula:

$$S(q) = \frac{\sum_{i=1}^{Nm} 2\sin(|q| * |r_i|)}{N_m} + 1 \qquad \text{(Equation S3)}$$

where $|r_i|$ denotes the distance between i-th pair of metal atoms and $|q|$ is the scattering vector length ($|q| = a2\pi/L$, where $a = 1, 2, 3\ldots\ q_{max} // q$).

The two-dimensional structure factor was calculated using the following formula:

$$S_{2D}(q) = \frac{\left(\sum_{i=1}^{Nm} \sin(q \cdot r_i)\right)^2 + \left(\sum_{i=1}^{Nm} \cos(q \cdot r_i)\right)^2}{N_m} \qquad \text{(Equation S4)}$$

where r_i denotes the distance vector between i- pair of metal atoms, and q is the scattering vector (q_x, $q_y = a2\pi/L$, where $a = q_{max} // 2q\ldots\ {-1}, 0, 1\ldots\ q_{max} // 2q$).

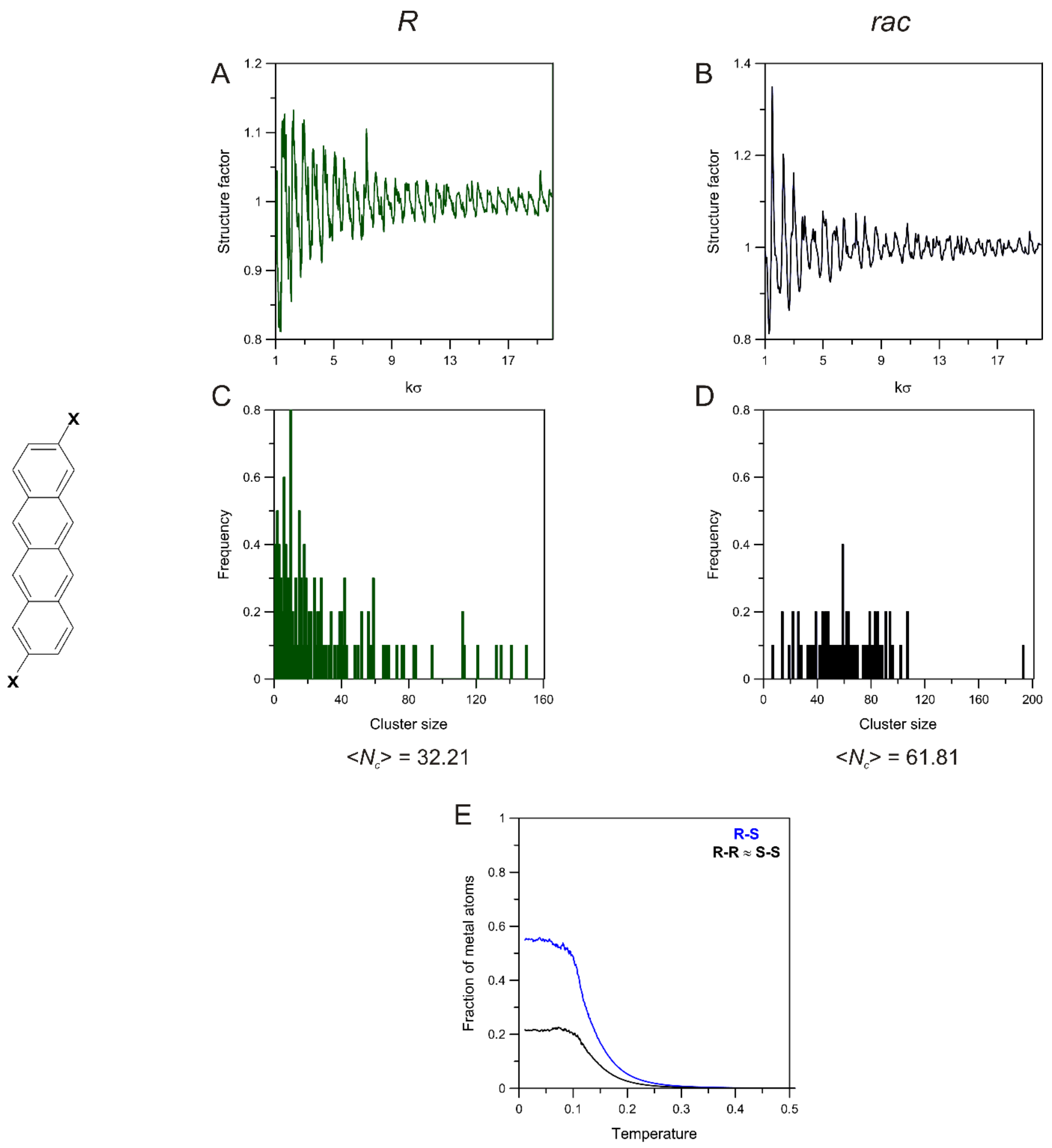

Figure S1. Structure factors (A, B), frequency of clusters of a given size and average size of the cluster (C, D) and fractions of homo- and heterochirally bonded metal atoms (E) for enantiopure (A, C) and racemic (B, D, E) systems of **t28** depicted in Figure 1.

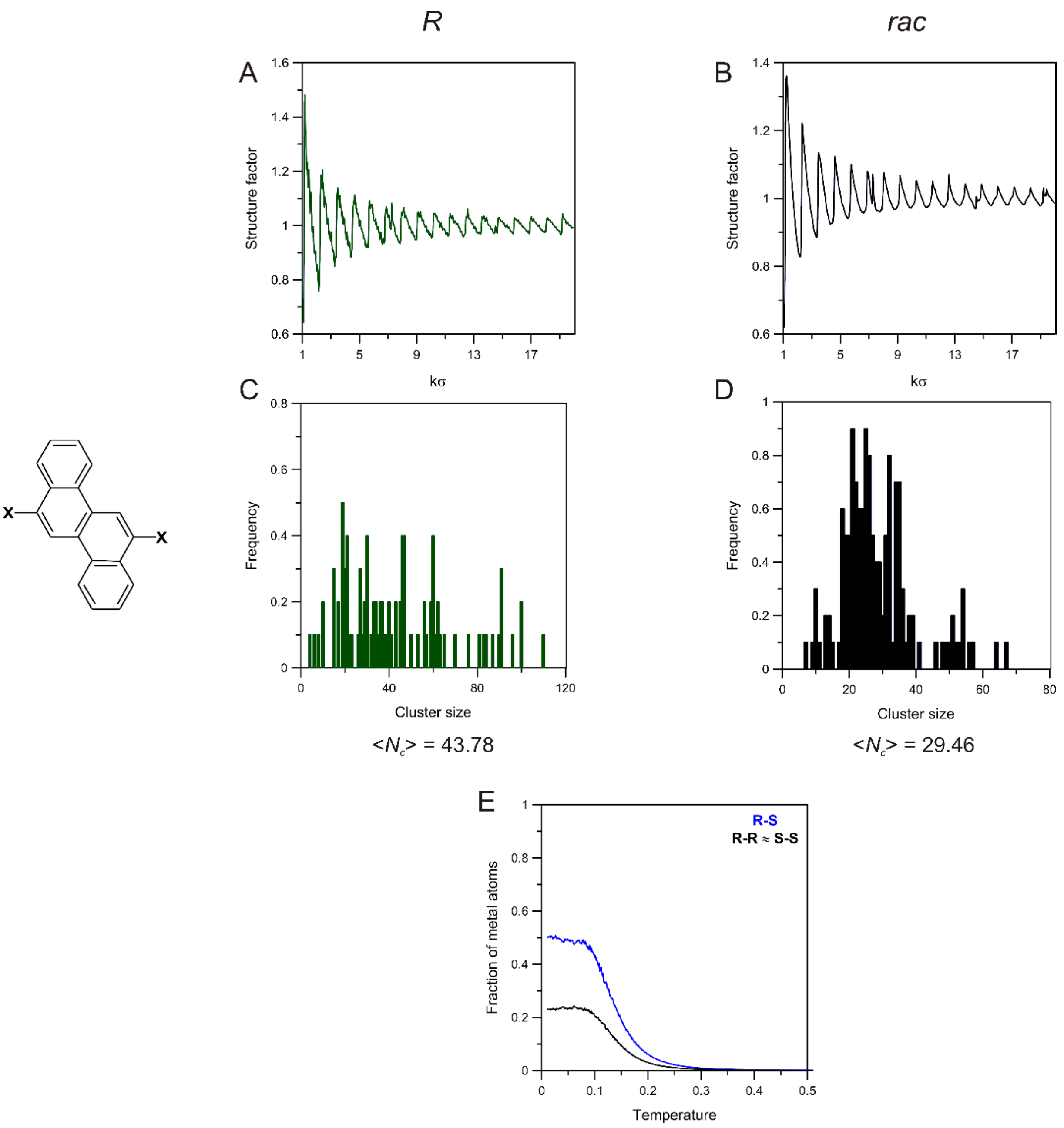

Figure S2. Structure factors (A, B), frequency of clusters of a given size and average size of the cluster (C, D) and fractions of homo- and heterochirally bonded metal atoms (E) for enantiopure (A, C) and racemic (B, D, E) systems of **c28** depicted in Figure 2.

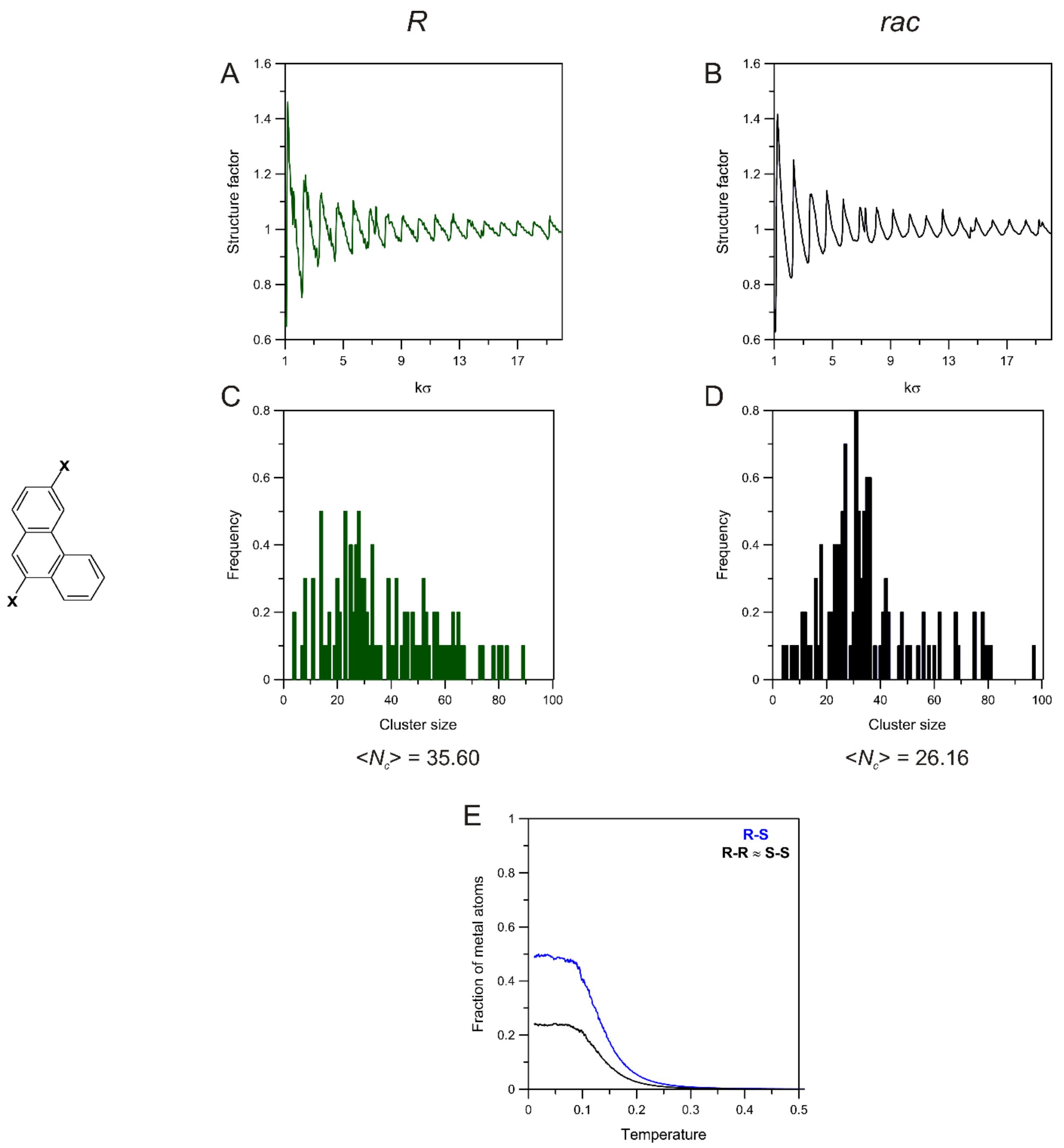

Figure S3. Structure factors (A, B), frequency of clusters of a given size and average size of the cluster (C, D) and fractions of homo- and heterochirally bonded metal atoms (E) for enantiopure (A, C) and racemic (B, D, E) systems of **p39** depicted in Figure 3.

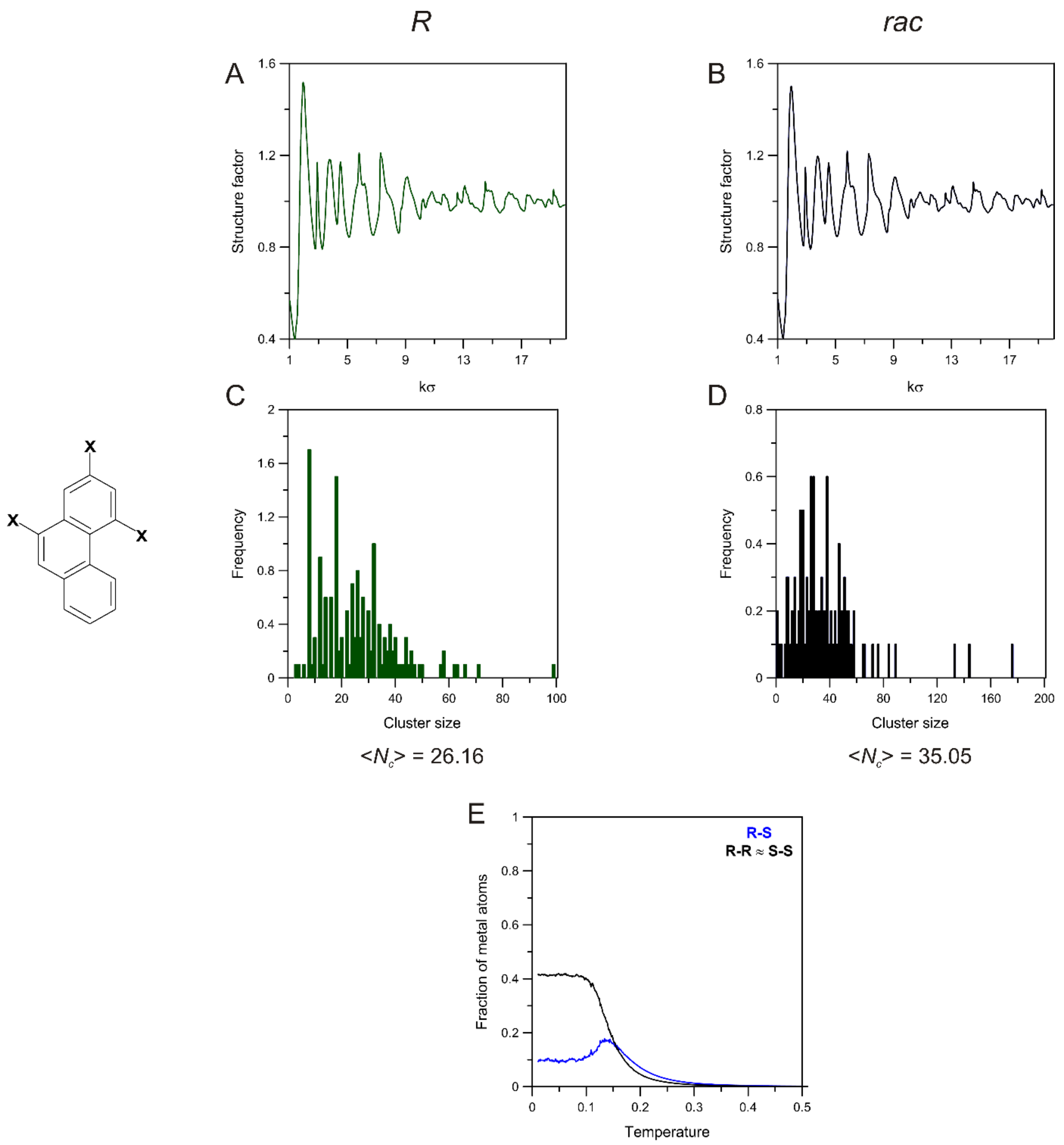

Figure S4. Structure factors (A, B), frequency of clusters of a given size and average size of the cluster (C, D) and fractions of homo- and heterochirally bonded metal atoms (E) for enantiopure (A, C) and racemic (B, D, E) systems of **p24_10** depicted in Figure 4.

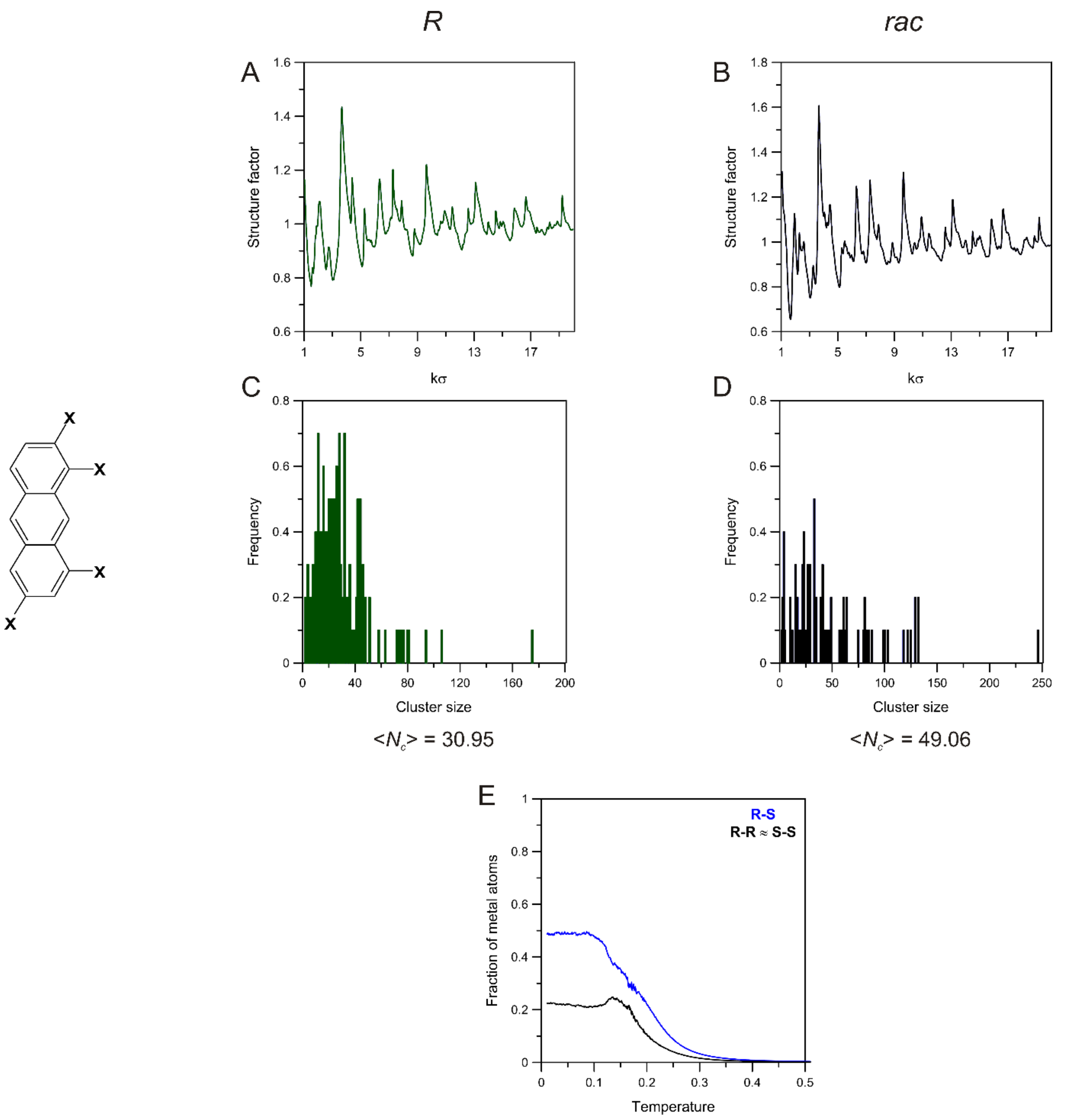

Figure S5. Structure factors (A, B), frequency of clusters of a given size and average size of the cluster (C, D) and fractions of homo- and heterochirally bonded metal atoms (E) for enantiopure (A, C) and racemic (B, D, E) systems of **a1268** depicted in Figure 5.

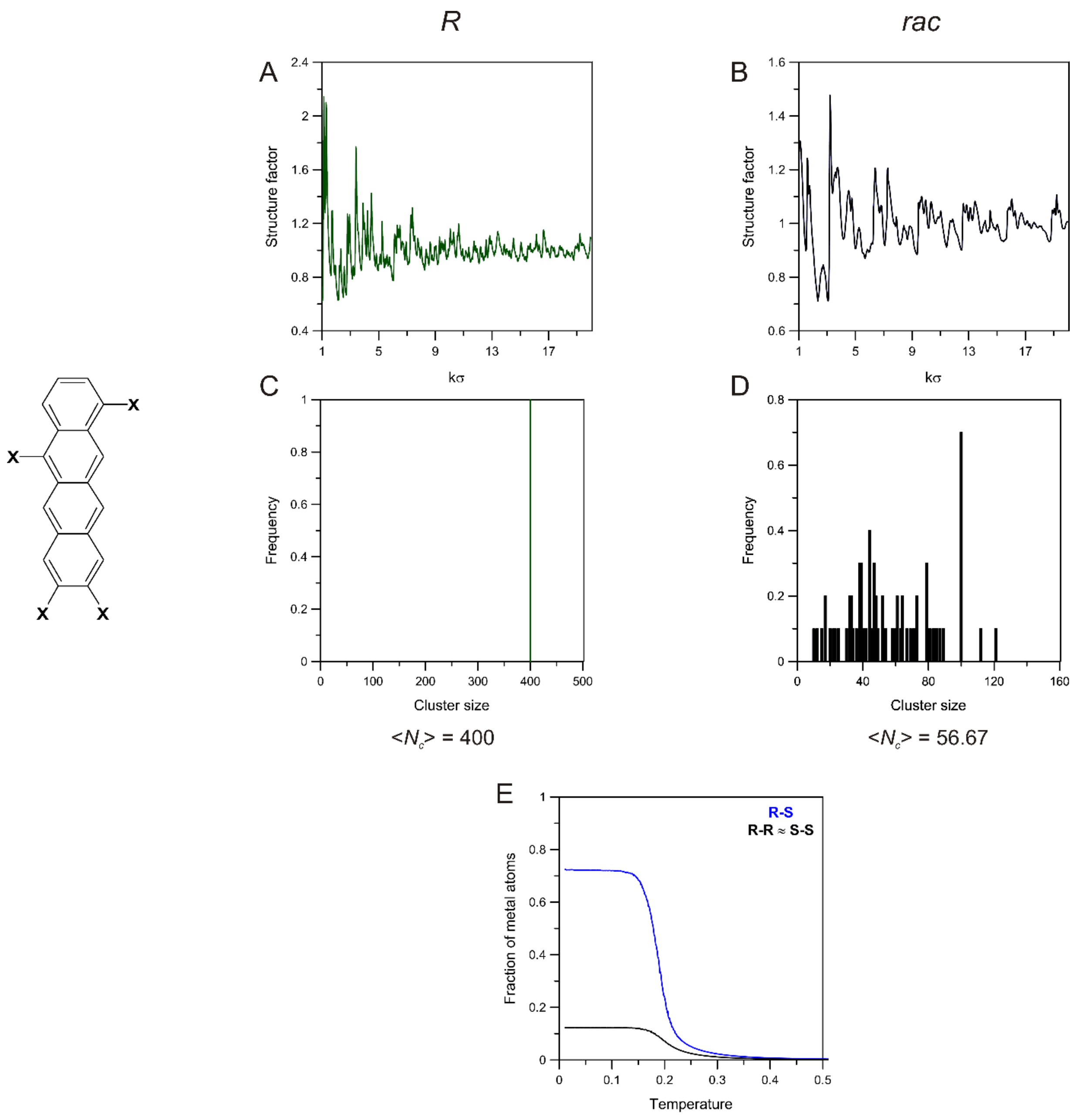

Figure S6. Structure factors (A, B), frequency of clusters of a given size and average size of the cluster (C, D) and fractions of homo- and heterochirally bonded metal atoms (E) for enantiopure (A, C) and racemic (B, D, E) systems of **t1589** depicted in Figure 6.
